# Impact of long-term daylight deprivation on retinal light sensitivity, circadian rhythms and sleep during the Antarctic winter

**DOI:** 10.1038/s41598-018-33450-7

**Published:** 2018-11-01

**Authors:** A. Kawasaki, S. Wisniewski, B. Healey, N. Pattyn, D. Kunz, M. Basner, M. Münch

**Affiliations:** 1 University of Lausanne, Hôpital Ophtalmique Jules-Gonin, Fondation Asile des aveugles, Lausanne, Switzerland; 2 Charité Universitätsmedizin Berlin, Institute of Physiology, Berlin, Germany; 3 Centre Hospitalier Alps Léman, 74130, Contamine-sur-arve, France; 4 Vrije Universiteit Brussel, Department of Human Physiology & Royal Military Academy, VIPER Research Unit, Brussels, Belgium; 5 St. Hedwig-Krankenhaus, Berlin, Germany; 6 Intellux GmbH, Berlin, Germany; 7 University of Pennsylvania, Department of Psychiatry, Perelman School of Medicine, Philadelphia, USA; 8 Charité Universitätsmedizin Berlin, Institute of Medical Immunology, Berlin, Germany

## Abstract

Long-term daylight deprivation such as during the Antarctic winter has been shown to lead to delayed sleep timing and sleep fragmentation. We aimed at testing whether retinal sensitivity, sleep and circadian rest-activity will change during long-term daylight deprivation on two Antarctic bases (Concordia and Halley VI) in a total of 25 healthy crew members (mean age: 34 ± 11y; 7f). The pupil responses to different light stimuli were used to assess retinal sensitivity changes. Rest-activity cycles were continuously monitored by activity watches. Overall, our data showed increased pupil responses under scotopic (mainly rod-dependent), photopic (mainly L-/M-cone dependent) as well as bright-blue light (mainly melanopsin-dependent) conditions during the time without direct sunlight. Circadian rhythm analysis revealed a significant decay of intra-daily stability, indicating more fragmented rest-activity rhythms during the dark period. Sleep and wake times (as assessed from rest-activity recordings) were significantly delayed after the first month without sunlight (p < 0.05). Our results suggest that during long-term daylight deprivation, retinal sensitivity to blue light increases, whereas circadian rhythm stability decreases and sleep-wake timing is delayed.

## Introduction

Compared with the long-term evolution of humans under natural light-dark cycles, the use of artificial lighting has – on an extremely short time scale - tremendously augmented and often replaced natural light. Artificial light is quantitatively and qualitatively different from natural light (e.g. brightness, spectral composition) and lacks variation related to geographical dissemination, seasonal change and the 24-hour light-dark cycle. All these effects impact not only on individual capabilities, but enable and foster large-scale societal activities and thus, the change to artificial lighting engenders a growing disconnection from nature^1,2^ and a fundamental cultural change. In the course of this still ongoing development, alterations of many biological, psychological and ecological conditions occur^3^ and concomitantly, various negative consequences on human health^4,5^ emerge. This will also have implications for normal and extreme working environments such as during space missions or on submarine ships.

Retinal phototransduction is mediated by the outer retinal photoreceptors (rods and cones) for visual perception and by the inner retinal photoreceptor (intrinsically photosensitive retinal ganglion cells ipRGCs) for mainly non-visual, light-dependent physiologic functions. One such function of ipRGCs is entrainment of innate biologic rhythms to the environmental light cycle. These innate rhythms have different period lengths, for example there is a circadian rhythm which cycles approximately every 24 hours and a circannual rhythm of about one year, and these rhythms largely govern the timing of most physiologic functions. Using the pupil as a ‘biomarker’ for both outer and inner retinal photoreception, we and others have shown that melanopsin activity but not visual photoreceptor sensitivity, varies between day and night time as well as between summer -winter season^6–9^.

Precedent individual light history gradually impacts the acute reactivity to light and the magnitude of its physiological and behavioural outputs, presumably by modulation of internal thresholds. For example, low light exposure during daytime sensitizes the system for light exposure in the evening as demonstrated by greater melatonin suppression in the evening when subjects had spent their preceding daytime hours in dimmer light^10–12^. In the same vein, exposure to low illuminance morning light over several days augments circadian phase shifts induced by (bright) light exposures in the evenings to a significantly greater extent compared to bright blue-enriched light exposure in the morning^13^. Low or aberrant light exposure during daytime thus may contribute to circadian misalignment, even in healthy persons not performing shift work.

There is clear evidence that changed light patterns (including those during space missions) also impact on mood and sleep^14–16^. Certain persons are vulnerable to the decreasing amount of daylight that occurs during fall and winter and as a consequence, develop a recurrent disorder of mood and behaviour consistent with clinical depression^17^. In these patients, recovery occurs spontaneously in late spring or summer or can be induced with light therapy. An animal model has reproduced pathologic behaviours of depression and anxiety in a nocturnal rodent by aberrant light exposures which recovered after administration of an antidepressive drug^18^. The exact mechanism by which light deprivation causes depression is not elucidated but may be, in part, related to suppression of the continuous production of new neurons in critical areas of the brain and alterations in central serotonin metabolism^19,20^.

Since electrical light exposure interacts and competes with natural light/dark cycles, total daylight deprivation in humans occurs in rare occupational situations such as prolonged submarine missions or space flight. Relative daylight deprivation, however, is relatively common and occurs namely at work places having reduced direct access to daylight such as coal mines, nuclear reactor facilities, library archives, indoor shopping malls, subway stations and even certain enclosed office spaces, surgical or intensive care units and laboratories. To better understand how retinal light sensitivity may be influenced by daylight exposure, the extreme seasons of the Antarctic or Arctic poles provide a natural setting of daylight exposure ranging from constant light during an extended period to no sunlight during the winter season. These remote lands are not habitable for long term human residence but research stations permit persons to work and live for periods of one year and longer. We have therefore chosen two stations in Antarctica (at different altitudes) to test the effects of long term daylight deprivation. The goal of this field study was to observe for changes in retinal light sensitivity in healthy humans before, during and after a protracted period of daylight absence. We also related the retinal light sensitivity to sleep parameters and circadian rhythms.

## Results

### Pupillography

#### Scotopic protocol

**Figure 1:**
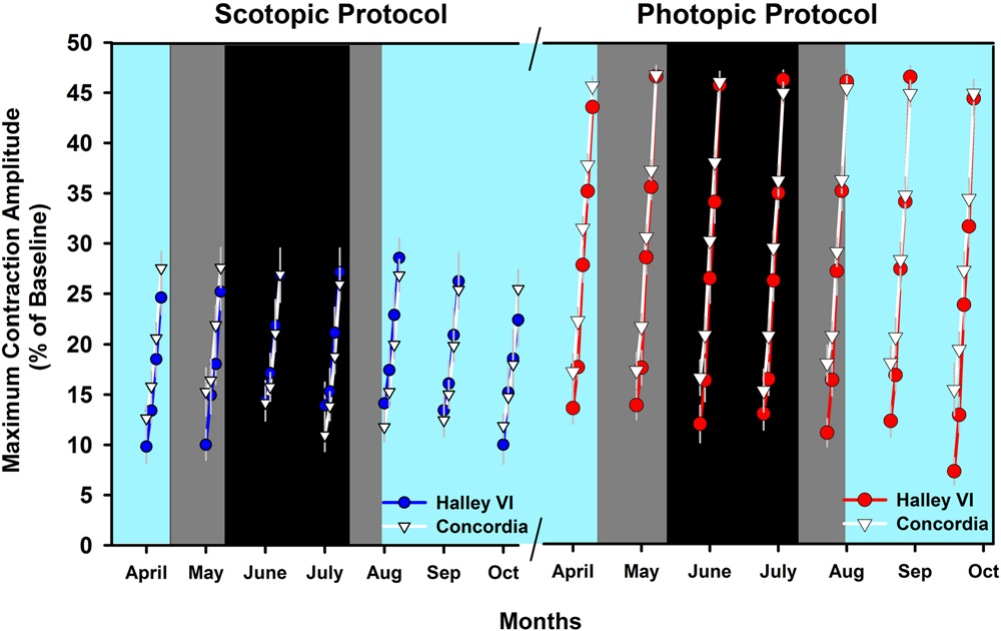
Maximum contraction amplitudes, averaged per condition and light stimulus for the rod-weighted protocol (scotopic; left) and cone weighted protocol (photopic; right). Red and blue filled circles depict averages for Halley VI and white filled triangles indicate results from Concordia (mean ± SEM). The cyan background indicates the weeks with direct sunlight (the data points are centered to the middle of each month), and the dark background is the period without direct sunlight. Within this period without direct sunlight, the black background indicates complete absence of sunlight (nautical/astronomical twilight) and the dark gray background indicates civil twilight (see legend of Supplemental Fig. S1 for definitions of civil, nautical and astronomical twilight).

In this protocol weighted to assess rod activity, the pupil response to four dim blue light stimuli (see method section and Supplement) presented following dark adaptation were compared between both stations and across 7 months: month 1 (=April): direct sunlight; months 2–4 (=May–July): no direct sunlight; months 5–7 (=August–October): direct sunlight again. There was a significant main effect of the factor LIGHT STIMULUS (F_3,66_ = 569.3; p < 0.0001), indicating (as expected) that maximal pupil contraction amplitude (CA) increases with increasing light stimulus intensity (Fig. 1, left graph). There was also a change over time across all light stimuli (main effect of TIME; F_6,122_ = 8.09; p < 0.0001) and a significant interaction with the factors STATION × TIME (F_6,122_ = 10.74; p < 0.0001), which revealed no difference between stations per month (p > 0.16), but a different time course of the change in pupil responses within each station when tested separately: Compared to April, Halley VI participants showed an increasing scotopic CA from June-October. The scotopic CA was significantly greater during the months without direct sunlight (i.e. May–July) and then decreased until October. Participants from Concordia showed a highest scotopic CA in May which subsequently decreased until October, with significant differences between May to July, September and October, as well as between June and July.

**Figure 2:**
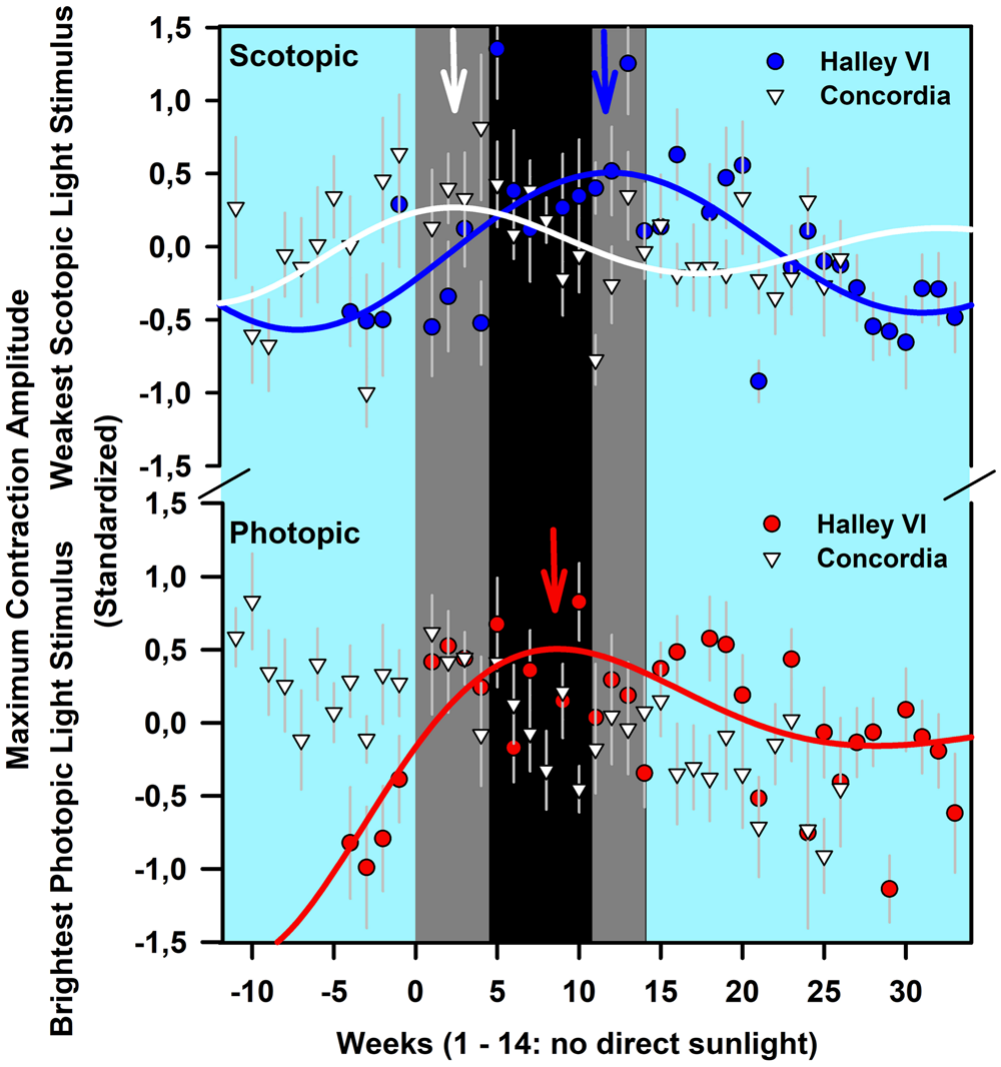
Maximum contraction amplitudes on standardized data (z-scores) for all weeks for the weakest light stimulus (−4 log cd/m^2^) of the rod-weighted protocol (upper graph; please note two upper error bars from Halley VI are partly cut) and the brightest red light stimulus of the cone weighted protocol (2.6 log cd/m^2^; lower graph). Red and Blue filled circles depict averages for Halley VI and white filled triangles indicate results from Concordia. The arrows indicate the greatest maximum contraction amplitudes (see text for exact hours and R^2^-values). The first week from Halley VI was excluded because it contained only one participant. Otherwise, means per station ± SEM are shown. The solid lines indicate the regression line from nonlinear sine fits. For the cone-weighted protocol there was a linear association for the Concordia station (not shown). The cyan background indicates weeks with direct sunlight, and the dark background is the period without direct sunlight. Within this period without direct sunlight, the black background indicates complete absence of sunlight (nautical/astronomical twilight) and the dark gray background indicates civil twilight (see legend of Supplemental Fig. S1 for definitions of civil, nautical and astronomical twilight). The blue, red and white arrows at the top indicate the week with the maximum contraction amplitudes (derived from fitted lines; see text for exact numbers).

When including all recorded weeks for the sine fitting for the dimmest of the four blue light stimuli (presumably the stimulus closest to threshold response under scotopic conditions), the peak response for Halley VI occurred at 11.9 weeks after the last sunrise (R^2^ = 0.47; p < 0.05) and for Concordia at 2.3 weeks after the last sunrise [R^2^ = 0.21; p < 0.05; Fig. 2, upper graph; standardized data (z-scores)]. This indicates an earlier increase of pupil responsiveness to very weak blue light stimuli in participants at Concordia compared to Halley VI.

#### Photopic protocol

The maximal, transient pupil contraction amplitude to the five bright red light stimuli of increasing intensity in this photopic protocol reflects predominantly M and L cone activity^21^. There was increasing photopic CA with increasing light intensity (main effect of LIGHT STIMULUS; F_4,88_ = 2238.3; p < 0.0001; Fig. 1, right). There was an interaction LIGHT STIMULUS × STATION (F_4,88_ = 15.1; p < 0.0001), but post-hoc tests corrected for multiple comparisons revealed no difference between both stations. The photopic CA significantly changed over time (main effect of TIME; F_6,123_ = 16.05; p < 0.0001) and there was a different time course of the photopic CA between both stations (interaction TIME × STATION; F_6,123_ = 3.59; p = 0.003). The photopic CA decreased significantly between April and October at both stations but at Halley VI, all months showed higher photopic CA compared to the very last month (i.e. October) whereas at Concordia, the photopic CA was higher only for the first three months (April–June) compared to the last two months (September–October).

When including all recorded weeks to the sine fitting for the brightest of the five red light stimuli (presumably the stimulus with minimal rod contribution and greatest M-L cone activation), there was a peak photopic CA for Halley VI at 8.7 weeks [R^2^ = 0.44; p < 0.05; on standardized data (z-scores)]. For Concordia, the same fitting curve did not converge to a nonlinear sine fitting but to a linear decrease (fit not shown; Fig. 2, lower graph).

#### Bright blue light protocol

**Figure 3:**
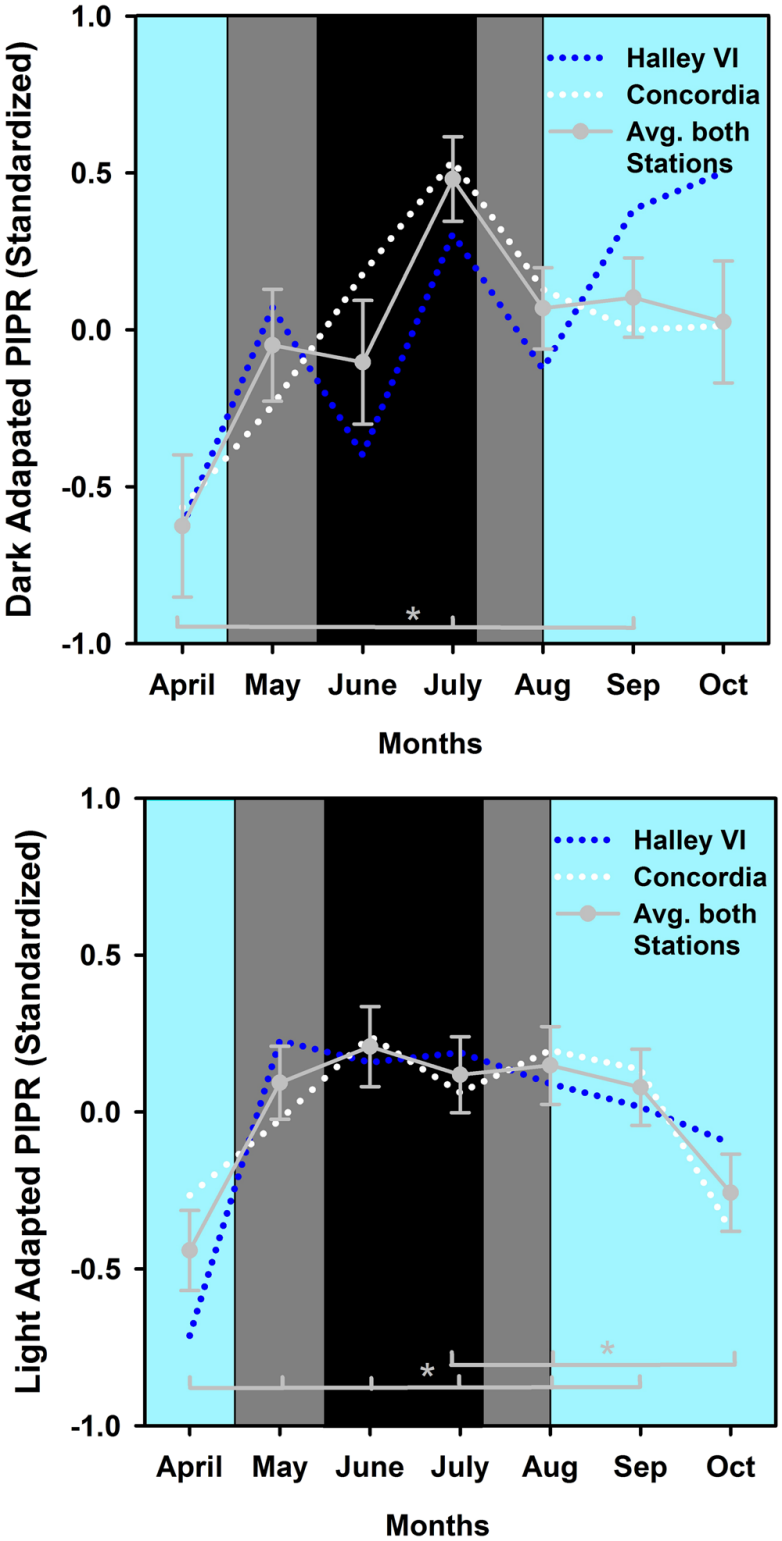
(**a**,**b**) Monthly bins for dark (**a**) and light adapted PIPR [(**b**); average of both light stimuli on standardized data (z-scores)]. The data is plotted per month and the months are labeled as approximate calendar months (the data points are centered to the middle of each month). The dotted blue line represents Halley VI and the dotted white Concordia station. The solid gray line and circles indicate the average of both stations and SEM (n = 24). The cyan background indicates weeks with direct sunlight, the dark grey areas indicate weeks without direct sunlight but civil twilight and the black area shows the time without direct sunlight and without civil twilight.

In a next step we analysed the pupil responses to bright blue light stimuli which increase the melanopsin contribution to the pupil response and can be evaluated from the post-illumination pupil response (PIPR, see method section). For equal light intensities, there was a significantly greater PIPR after dark adaptation, as has been shown previously^21^ (main effect of LIGHT STIMULUS; F_1,22_ = 1258.37; p < 0.0001) and further comparisons were done on standardized data (z-scores). For the dark adapted PIPR alone, there was a main effect of TIME such that the PIPR significantly increased between April and July and between April and September (Fig. 3a; F_6,122_ = 5.5; p < 0.0001). There was no difference in PIPR between stations or an interaction with the factor STATION (p > 0.3).

**Figure 4:**
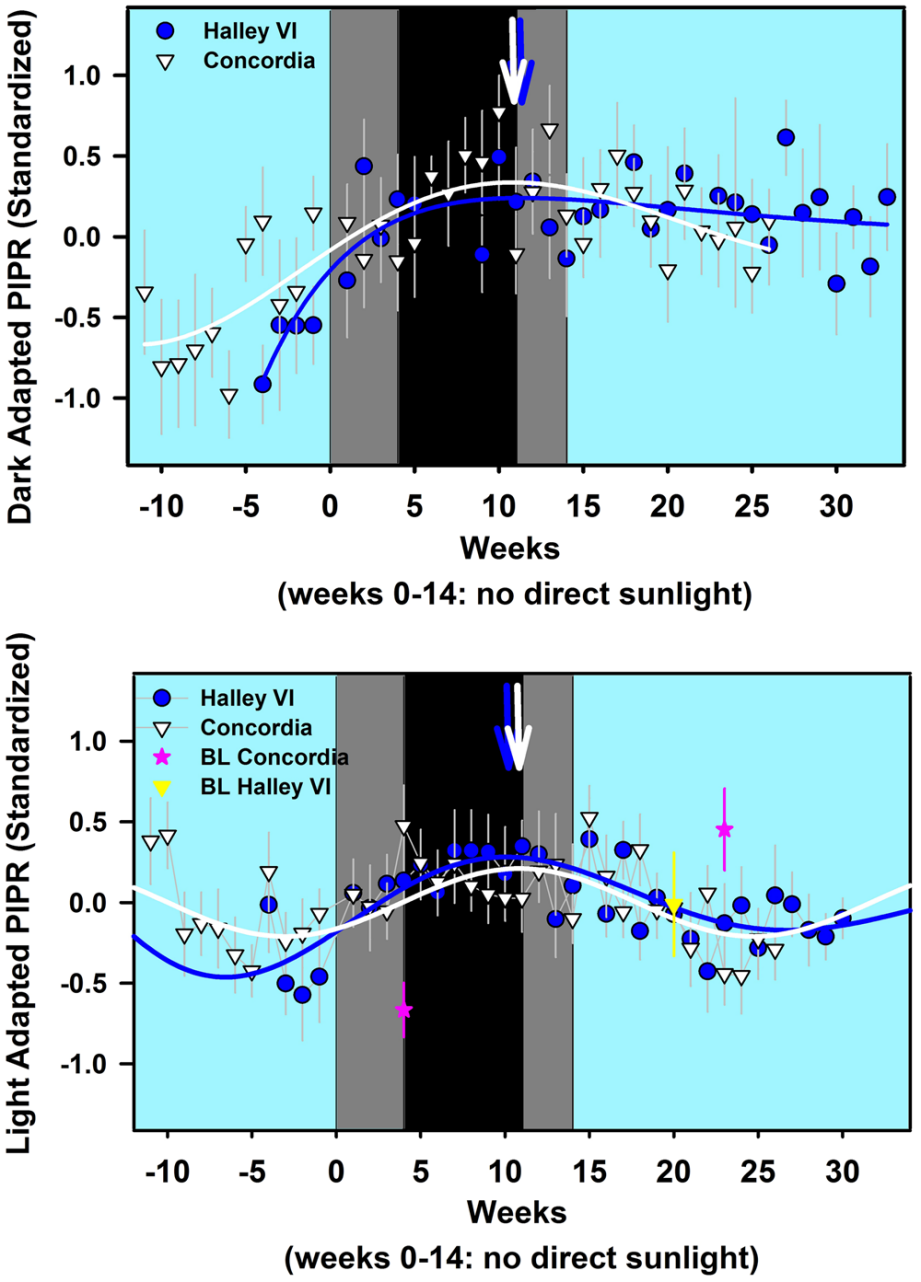
(**a**,**b**) Nonlinear curve fits for dark adapted PIPR **(a)** and light adapted PIPR **(b)** across all week on standardized data (z-scores) as a function of time for both stations (Halley VI: solid blue line; Concordia: solid white line; n = 24) Blue and white symbols indicate weekly averages for both stations (± SEM). The pink stars indicate baseline values for Concordia, taken during the pre-and post mission measures and the yellow triangle indicates the pre-mission measures for Halley VI (no post-mission recordings, see methods). The blue and white arrows at the top indicate the week with the maximum PIPR (derived from fitted lines; see text for exact numbers). The cyan background indicates weeks with direct sunlight, the light grey areas indicate weeks without direct sunlight but civil twilight and the black area shows the time without direct sunlight and without civil twilight.

The non-linear fitting of the dark adapted responses (all weeks) revealed a maximum PIPR in Concordia participants (R^2^ = 0.65) after 10.8 weeks since the last sunrise, and in the Halley VI participants (R^2^ = 0.62) after 11.3 weeks (Fig. 4a). The nonlinear fitting of dark adapted PIPR for Halley VI participants was performed without weeks 6 and 7 where the PIPR was more than 2 SD lower than the preceding and following weeks (outlier) and without the fifth week before the last sunrise, where only 1 participant was tested.

We compared the PIPR in response to two different blue light intensities recorded under photopic conditions (see method section). The PIPR to the first light stimulus was very small (4.4% ± 3.7% across all months and for both stations) and was significantly smaller than PIPR determined from the second brighter blue light stimulus (p < 0.0001). We then used the average of both light adapted light stimuli on standardized data (z-scores; Fig. 3b). There was a main effect of TIME (F_6,123_ = 5.91; p < 0.0001) with a significantly lower PIPR in April than May to September and a lower PIPR in October than in July and August. There were also significant interactions between STATION × TIME (F_6,123_ = 2.49; p = 0.026) and STIMULUS × TIME (F_6,122_ = 2.43; p = 0.03) but post-hoc tests were not significant after correction for multiple comparisons (see method section). There were no significant differences for the covariate BL pupil size (p = 0.3). The significant covariate age indicated smaller PIPR for older crew members (p = 0.0003).

The nonlinear sine fits using all recorded weeks (except for the first during which only 2 participants were tested) from both stations show that the maximum photopic PIPR occurred within the same week at both stations (week 10.1 after the last sunrise for Halley VI; R^2^ = 0.59; and in week 10.8 after the last sunrise for Concordia; R^2^ = 0.35; p < 0.05). Baseline data were also added for both stations at the approximate week when they were taken. For the Concordia station, post-mission measures showed a significant decrease in PIPR compared to those recorded in the first month without direct sunlight (=May; p < 0.05; Fig. 4b).

#### Baseline pupil sizes

For mean values and statistics of baseline pupil sizes for each protocol see Table S1 and text in the Supplemental Material. Correlations between the baseline (BL) pupil sizes and the scotopic CA (Spearman Rho = −0.33) and the photopic CA (Spearman Rho = −0.22) revealed significant negative correlations (p < 0.001) such that larger BL pupil sizes were related to smaller CAs. For the dark and light adapted PIPR (average of both light stimuli for the light adapted PIPR) there were no significant correlations between BL pupil sizes and the PIPR (p > 0.2).

### Circadian rhythm analysis

**Figure 5:**
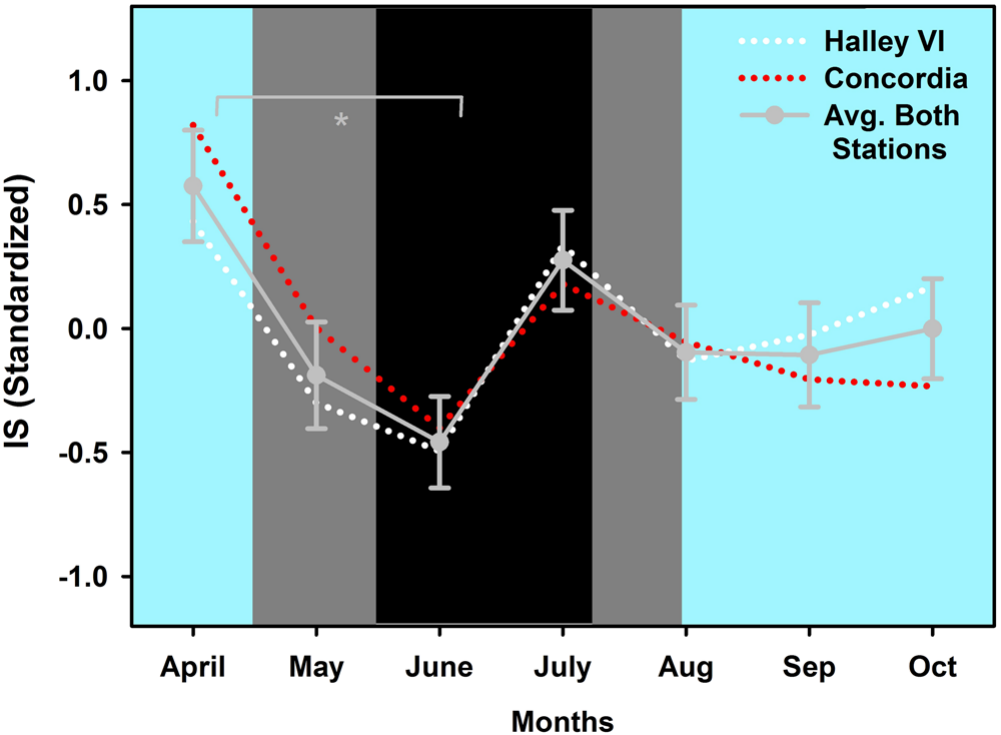
Inter-daily stability for both stations on standardized data (z-scores; white dotted line: Halley VI; red dotted line: Concordia) and averaged across stations (grey solid line and grey circles; ±SEM) across 7 months. *Significant main effect of TIME between month 1 (April) and month 3 (June; p < 0.05). The cyan background indicates weeks with direct sunlight, the grey areas indicate weeks without direct sunlight but civil twilight and the black area shows the time without direct sunlight and without civil twilight.

**Figure 6:**
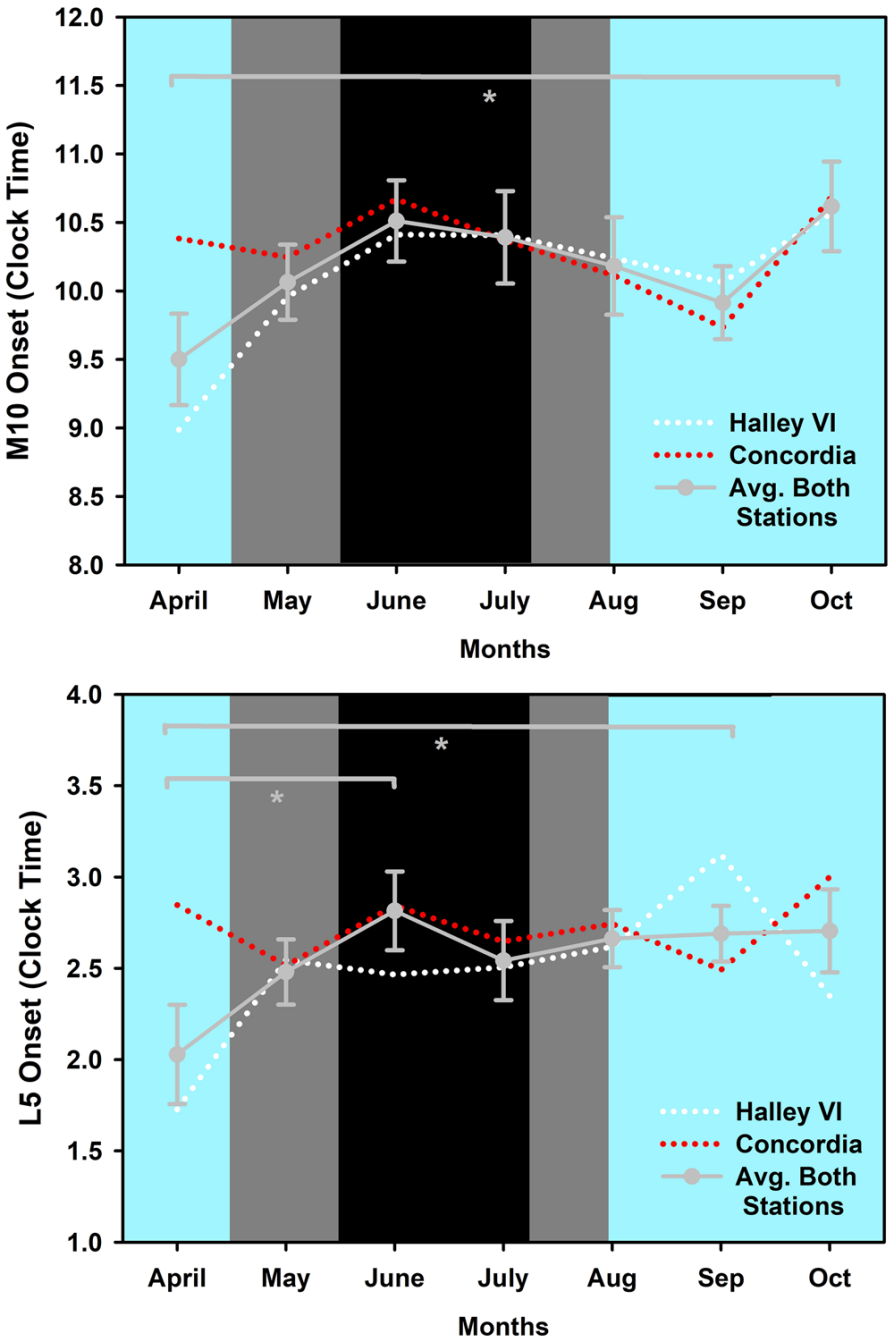
(**a**,**b**) These Figures show the time onset for the 10 hours with highest activity (M10on; **a**) and the time onset for the 5 hours with lowest activity (L5on; **b**). Those are shown in clock time and averaged per month for both stations (means, SEM).

In order to compare the time course of rest-activity cycles with the pupil data, we used the same time range of data for both stations and collapsed them in monthly bins (except for the first month, where we included 5 weeks for the circadian rhythm analysis). This resulted in 7 months for analysis (one month before = April, three months during May-July, and three months after the time without direct sunlight August-October). Rest-activity data from one participant at Halley VI contained only seven weeks, and recordings of five participants from Concordia could not be used because either the data was not available or the data was insufficient from not wearing the watch at night time. A total of 74.2% (3017 days; 1760 days from Halley VI; n = 12; 1257 days from Concordia; n = 8) of all edited rest-activity data (4064 days) was used for analysis (~7 months). There was a significant decline of inter-daily stability (IS; see methods) after two months without direct sunlight [main effect of TIME; F_6,99_ = 2.2; p = 0.0496; Fig. 5; on standardized data (z-scores)]. There was also a later onset of the ten-hour period with greatest activity (M10on) across time (main effect of TIME; M10on; F_6,99_ = 2.45; p = 0.03; Fig. 6a), such that the time of M10on was 1:07 h later at the end of three months after the sun came back (October) than in April. The timing of the five-hour period with lowest activity (L5on) went in the same direction (main effect of TIME; L5on; F_6,99_ = 3.16; p = 0.007; Fig. 6b): the onset time of L5 became significantly later between the month before the last sunset (April) and the second month without direct sunlight (June) as well as between the first (April) and the second to last month (September). None of the other tested variables [intra-daily variability (IV), amplitude of the 10 h with greatest (M10), and amplitude of the 5 h with lowest activity (L5), relative amplitude (relamp; ratio of M10/L5)] showed a significant change over time (main effect of TIME; p > 0.05), and there was no difference between the two stations (p > 0.04) or an interaction between these factors (p > 0.09) except for L5 (STATION × TIME; F_6,99_ = 2.27; p = 0.043) or an effect of age (on absolute values) or sex. However, after correction for multiple comparisons (false discovery rate, see methods) none of the post-hoc tests remained significant.

Correlation of the circadian variables (IS, IV, M5, M5on, L5, L5on, relamp) with the PIPR revealed no significant correlations for any of the variables (p > 0.08).

### Sleep (derived from rest-activity recordings)

For sleep episodes (derived from activity recordings) there was a main effect of TIME for bed and wake time, time in bed, sleep start and end, and sleep duration (F_6,103_ > 3.8; p < 0.05), whereas the other variables [percentage of sleep duration, wake time and percentage of wake time, sleep efficiency (sleep time/time in bed*100), sleep and wake bouts; see Table 1] did not significantly vary over time. Bed- and wake times gradually moved to later times after the first month before the overwinter season until the first two months without direct sunlight (May-June) and also between April and August-September (i.e. April vs. August for bed times, and between April-May vs. August-September for wake times). Time in bed became longest in October, i.e. 3 months after the return of direct sunlight compared to all other months except the first month (April; p = 0.002). Compared to the first month, sleep start and end gradually occurred later during the mission: the differences became significant between April and June, May and June, as well as between April and July/August for sleep start. For sleep end, the differences were significant between April and June/September/October as well as between May and October (main effect of TIME; p < 0.05). Sleep duration was longest in the last recorded month (October) when compared to all other months. The younger subgroup had significantly more sleep and wake bouts than older (covariate AGE; p = 0.02). There was no difference for any of the time points between both stations (p > 0.07).

**Table 1:** Sleep variables derived from rest-activity recordings.

Month	1 (April)	2 (May)	3 (June)	4 (July)	5 (Aug)	6 (Sep)	7 (Oct)
Bed Time*	0:41	1:07	1:42	1:10	1:11	1:17	1:09
(SD)	(2:07)	(1:41)	(1:53)	(1:42)	(1:33)	(1:34)	(1:40)
Wake Time*	8:25	8:51	9:15	8:50	8:53	9:02	9:12
(SD)	(1:49)	(1:32)	(1:49)	(1:35)	(1:28)	(1:29)	(1:46)
Time in Bed*	7:47	7:43	7:33	7:40	7:42	7:45	8:02
(SD)	(1:32)	(1:37)	(1:36)	(1:34)	(1:29)	(1:34)	(1:38)
Sleep Start*	0:44	1:14	1:48	1:16	1:17	1:24	1:16
(SD)	(2:00)	(1:40)	(1:53)	(1:42)	(1:33)	(1:34)	(1:40)
Sleep End*	8:24	8:50	9:15	8:50	8:53	9:01	9:11
(SD)	(1:49)	(1:32)	(1:48)	(1:34)	(1:28)	(1:29)	(1:46)
Sleep Duration*	7:09	7:07	6:57	7:03	7:06	7:08	7:24
(SD)	(1:26)	(1:29)	(1:29)	(1:28)	(1:22)	(1:28)	(1:33)
Sleep Duration (%)	93.38	93.53	93.57	93.45	93.58	93.57	93.53
(SD)	(2.57)	(2.66)	(2.79)	(2.56)	(2.32)	(2.48)	(2.37)
Wake Time	0:30	0:29	0:28	0:29	0:29	0:29	0:30
(SD)	(0:13)	(0:14)	(0:13)	(0:13)	(0:12)	(0:13)	(0:12)
Wake (%)	6.62	6.47	6.43	6.55	6.42	6.43	6.47
(SD)	(2.57)	(2.66)	(2.79)	(2.56)	(2.32)	(2.48)	(2.37)
Sleep Efficiency (%)	91.91	92.09	92.02	91.91	92.07	92.04	92.06
(SD)	(3.26)	(3.17)	(3.27)	(2.90)	(2.70)	(2.93)	(2.75)
Sleep Bouts (#)	20.25	19.35	18.74	19.45	19.08	19.67	19.88
(SD)	(8.58)	(8.85)	(7.92)	(8.08)	(7.17)	(8.08)	(7.92)
Wake Bouts (#)	20.15	19.24	18.63	19.35	18.94	19.49	19.73
(SD)	(8.58	(8.87)	(7.87)	(8.04)	(7.20)	(8.07)	(7.93)

Sleep variables are averaged for both stations (Standard deviations = SD; in the respective cell below, in brackets) since there was no significant difference between both stations; n = 20. Data is shown as clock time (hours:min) for bed and wake time, sleep start, sleep end, and sleep duration. Wake, sleep efficiency are shown in percentage (%) and sleep and wake bouts are indicated in absolute numbers per night (#). *p-value < 0.05 for significant main effect of TIME (months 1–7). For description of the significant differences between months see text.

Correlations of the sleep variables with the PIPR revealed small but significant associations between a greater PIPR and less time in bed, shorter sleep duration and more sleep and wake bouts (Spearman Rho range: −2.4 to 1.9; p < 0.04).

## Discussion

This study examined the effect of long-term daylight deprivation on retinal sensitivity, circadian rhythms and sleep parameters before, during and after the Antarctic winter when there is no direct sunlight for 3.5 months. Retinal sensitivity was represented by the pupil responses to different light stimuli which is not an ideal assumption but served as a proxy during the mission on two different Antarctic stations. On the whole, we found that retinal sensitivity increased following the disappearance of daylight. This increased sensitivity to acute light stimuli was indicated from the pupil responses to scotopic, photopic and bright blue stimulus protocols and was most clearly observed with the PIPR, the biomarker used in this study for melanopsin sensitivity.

From the photopic pupil responses to red light stimuli, our marker of cone sensitivity, there was variable light sensitivity during the period without direct sunlight. The ability of cones to cope with fast light changes across a broad spectrum of illuminance may have influenced adaptive changes to long term daylight modulation. Scotopic pupil responses to dim blue lights, presumably a global measure of rod sensitivity, showed increasing contraction amplitude until a maximum was reached at 2.3 weeks after last sunrise for Concordia participants and at 11.9 weeks for Haley VI participants. After reaching peak sensitivity, there was subsequent decline in rod sensitivity, occurring earlier for Concordia participants compared to those at Halley VI (Fig. 2). What might account for the differences in the time course of changing rod sensitivity between these two stations? One possible explanation could be differences in their daylight pattern. The two Antarctic stations, though situated at the same geographical latitude, do not have the same longitude. However, analysis of the exact timing and duration of daylight from direct sunlight to civil twilight to darkness (i.e. in nautical/astronomical twilight) revealed very minor differences. For example, the day with the last sunset was shifted only by 2 days between Concordia and Haley VI and duration of civil twilight was similar between the two stations (see Supp. Fig. S1). Thus, differences in daylight cannot explain the difference in the time course of changing rod sensitivity between the two stations. Another possible influence is altitude. The higher altitude of Concordia may have a stronger influence as atmospheric air pressure is much lower. Higher altitude has been shown to alter pupil responsiveness to dark adaptation and result in larger contraction amplitudes^22,23^, longer latency times and smaller baseline pupil sizes^23,24^. However such changes are transitory and adapt after several days of acclimatization^24^. In our study, the absolute baseline pupil size and the magnitude of pupil contraction were not different between stations and we therefore assume that Concordia participants were acclimatized to their higher altitude.

We recognize that dark and light adaption times used in this study are shorter than standard recommendation and as such, prior light exposure (indoor and daylight) might be a confounding influence. For example, participants working at Halley VI station which has brighter artificial indoor lighting (see Suppl. p. 2–3) may have less retinal effect from 10 min dark adaptation on rod sensitivity.

These differences in daily (room) light intensities between both stations did not appear to affect baseline pupil size or responses in the scotopic and photopic condition since there were no statistical significant differences in any of the four baseline pupil sizes of the scotopic and the five baseline pupil sizes of the photopic protocol between both stations for any of the months. In the same vein, there was no statistical significant difference of the contraction amplitudes to different light stimuli of the scotopic and photopic protocol between both stations, suggesting that participants at the two stations were similarly dark and light adapted, even though we did not measure individual light exposure recordings over the mission.

Despite differences in artificial room lighting between both stations, the main difference is that during the dark months all participants were in a more dark-adapted state prior to testing because of the lack of direct sunlight. Therefore, retinal sensitivity was greater after 10 min dark adaptation for all participants when compared to dark adaptation prior to pupil tests in April or October, where they had been in brighter environments (i.e. with direct sunlight) prior to testing. Our results most likely reflect the interaction of short- and long term adaptation effects to light which changed in the course of the mission.

The pupil response, specifically the PIPR, to the bright blue light protocol was used in this study as a proxy for melanopsin sensitivity to acute light stimulation repeatedly measured over several months. The first change that emerged as direct sunlight and daylight duration waned and then disappeared was a progressive increase in PIPR from the first month of darkness to a maximum PIPR at 10 weeks after last sunset. This time course of the increasing PIPR was observed for both dark-adapted and light-adapted PIPR and for both stations. We presume this increased PIPR reflects increased melanopsin sensitivity. The PIPR did not begin to decrease until two months after the sun came back. The overall pattern of changing PIPR over 7 months resembles an inverted U in which inner retinal light sensitivity increases rapidly when daylight extinguishes but is slower to return to pre-darkness levels. Whether this adaptation occurs at the level of retinal phototransduction or post-synaptically cannot be determined from this study. Our PIPR results are consistent with an earlier study performed during the Antarctic winter. The authors reported stronger melatonin suppression by light during the Antarctic winter than during the summer season^25^ and concluded that retinal sensitivity to light may increase during the overwinter season.

The steady increase in baseline pupil size over the course of the mission (see text and Supplemental Table S1) seems unrelated to environmental light conditions. Also the continuous increase of baseline pupil size occurred in both stations similarly, and would thus appear unrelated to altitude differences between the stations. We can only presume that other supranuclear influences resulting in decreased inhibition of the Edinger-Westphal nucleus play a role. The increasing baseline pupil size does not explain the changing (increasing-decreasing) pattern of scotopic and photopic pupillary contraction amplitude and the dark- and light-adapted PIPR observed over the course of the mission.

The long-term daylight deprivation on sleep and circadian rest-activity cycles revealed greatest effect on the inter-daily stability, a proxy for circadian fragmentation. The IS decreased during the first two months without direct sunlight in parallel in both stations. A greater circadian fragmentation has been described in some^26,27^ but not all studies^28^ performed in the extreme environments such as the Arctic/Antarctic poles.

Some studies also showed that sleep was more affected during the summer season (i.e. 24-h daylight) with shorter sleep duration^29^, and less slow wave and rapid eye movement sleep^30^. The sleep timing delay we found in our study, again for both stations, occurred continuously over the entire time of the mission and this has been described repeatedly by other studies from confined and isolated environments^31–35^, even when individual light exposure was increased^31^. To summarize, our sleep and circadian results during the overwinter period may partly be caused by the lack of daylight, but also by the isolated and confined environmental conditions and work schedules (for a review see^36^).

We recognize that, as a field study, the major limitation stems from the lack of control for many variables which might influence retinal light sensitivity and/or circadian rest-activity cycles. Such variables include duration and type of daily light exposure (both artificial and daylight), kinds of daily activity, track records of social interactions, eating habits and work schedules. Though these variables were not controlled our results are, nevertheless fairly similar for the two stations, and in particular there is a striking similarity of the time course of changing PIPR response.

Taken together, our results demonstrate increased outer and inner retinal sensitivity to acute light stimulation with progressive loss of daylight. Our results add further evidence that despite the modern lifestyle in which exposure to artificial lighting prevails over natural light, there is an impact of long-term light history on human physiology. In this study, the increased retinal sensitivity was best observed in the post-illumination pupil response, implicating the melanopsin system. Why might this be interesting? Melanopsin has been implicated in light-dependant changes in behaviour in rodents^18^ reminiscent to depressive states in humans. Decreased retinal light sensitivity assessed by the pupil response was shown for patients with seasonal affective disorder^37^ and in patients with non-seasonal depression^8^. The pupil response to light might serve as a marker for identifying persons vulnerable to depression or other diseases where melanopsin is implicated.

Another population where our results may have significance is persons working in extreme environments such as space stations and long-term submarine missions. In these conditions, appropriate artificial light exposure is needed to maintain synchronization of the circadian clock. Our finding of increased instability of circadian rest-activity cycles which manifested during the first two months of polar winter suggests that brighter artificial (indoor) light may be helpful in offsetting circadian fragmentation, as it was shown previously^38,39^. Additionally, the increased retinal sensitivity during daylight deprivation might make such persons more vulnerable to light exposure in the evening and lead to, for example, greater melatonin suppression at night and further difficulty with sleep and circadian synchronization. It is clear that further studies examining effects of long-term light history, daily light exposure and acute light stimulation on human physiologic systems are needed to help us understand and optimize the artificial and natural light conditions best suited for healthy living in modern times.

## Methods

### Study site

The study was conducted on two different Antarctic stations during the overwinter season of 2015. One station was Halley VI (operated by the British Antarctic Survey, UK). This station is situated at sea level and located 26°39′ West and 75°35′ South. The other station was Concordia (operated by France/Italy, and used by the European Space Agency as research facility) which is located 3200 m above sea level and approximately at the same geographical latitude: 120°20′ East and 75°06′ South. The climate at both stations is extreme: temperatures in winter go below −80 °C (Concordia) with extremely low humidity and low air pressure. At both study sites, there is no direct sunlight for approximately 105 days per year with different twilight levels during this time (for detail see Supplemental Material and also Fig. S1). The working routines consisted of various duties, such as station maintenance work (heating, electricity, water, and food supply), scientific measures of the atmosphere and ice, collection of environmental samples and participation in different biomedical and psychological tests as participants for several international research projects. We confirm that all research was performed in accordance with the principles of good scientific practice at Charité University Medicine Berlin, Germany.

### Participants

Twenty-five participants i.e. all members from both crews (Halley VI: 4 women/8 men; Concordia: 3 women/10 men) were included in the study. Written informed consent of all participants was obtained before the study started and all procedures were approved by the local Ethical Review Board (Charité University Medicine Berlin, Germany). All crew members were familiarized at least 3 months in Europe prior to the mission. The mean age of participants from Halley VI was 33.7 ± 11.2 yrs (±SD; range: 22–58 yrs) and from Concordia 34.6 ± 11.0 (range: 25–56 yrs). Participants had a thorough medical screening procedure before the mission; organized by the European Space Agency (for Concordia) and the British Antarctic Survey (for Halley VI; details for the screening questionnaires of both stations can be found in Supplemental Material and Table S2).

### Pupil recordings

The pupil responses were recorded with a portable pupillometer (Neurolight, IDMed, Marseille, France) at a sampling frequency of 67 Hz. Recordings were taken weekly from February through October 2015 (Concordia) and from April through December 2015 (Halley VI), resulting in a recording overlap of 7 months for both crews. The pupil recordings were performed by the same person at each station and always during waking hours between 8:00 and 18:00 local time. Total testing time was about 25 min, including 10 min of dark adaptation and 10 min of room light adaptation (see below and Supplemental Material for light stimuli protocols). As participants are crew members whose principal responsibility is the maintenance of the station during the overwinter period, a longer period of dark or light adaptation was considered disruptive to the work schedule and thus not authorized by the station administrators. For all but two participants (see Supplemental Text), the pupil recordings were taken on the right eye. After each recording, pupil tracings were downloaded, coded and saved locally on a PC. The data was later sent to Germany for analysis.

All recorded tracings were visually inspected by a trained assistant and artifacts induced by movement and/or blinking were removed by linear interpolation. From 2350 tracings, 66 (2.8%) had to be excluded due to poor quality. Tracings were normalized by expressing absolute pupil size in mm (PS) as a percentage of baseline pupil size in mm (BL). BL was calculated as the averaged pupil size during 0.25 s before each light stimulus. Relative pupil size (RPS) at any given moment was assessed as pupil size relative to baseline pupil size in percentage: RPS = (PS/BL * 100). For the tracings obtained with the scotopic and photopic test protocols (see Supplement), the maximum contraction amplitude (CA) was the main outcome parameter and calculated as: 100% (=BL) - minimum RPS. Large contraction amplitudes indicate a strong pupil contraction in response to a light stimulus. For the bright blue light protocol, the post-illumination pupil response (PIPR) was the main parameter and calculated as: 100% (=BL) - RPS at 6 s after the light stimulus offset. A large PIPR depicts a sustained pupillary constriction and delayed re-dilation of the pupil after the offset of the light stimulus.

### Light stimulus for pupillography

Three different light sequences (protocols) for pupil testing were consecutively executed for each participant every week. The protocol started after 10 min of dark adaptation with four weak blue-light stimuli under scotopic conditions (rod-weighted; for details of the light stimuli protocols see text in the Supplemental Material, pages 3–4), followed by one bright blue light stimulus (dark adapted, melanopsin weighted). The participants where then light-adapted to room light for 10 min and underwent the photopic pupil testing with a series of 5 bright red light stimuli (L-/M-cone-weighted), and two bright blue light stimuli of different intensities (melanopsin-weighted).

### Circadian rhythm analysis and sleep (derived from activity watches)

Rest-activity cycles were recorded before, during and after the overwinter season by using wrist-worn activity watches (Motion Watch 8, Camntech, Cambridge, UK and Actigraph, Actigraph GTX9 Link, FL, USA). During the mission, rest-activity recordings were regularly downloaded to a laptop. Single data files were joined post-mission to one file per participant. Each 24-h recording was visually inspected in order to edit or exclude days: days with more than 3 h of missing data during daytime were excluded from the analysis. Missing data which were shorter than 3 h were edited with the mean activity of 24 h by using the software Sleep Analysis (v7.2, Camntech, Cambridge, UK). Edited rest-activity recordings underwent a nonparametric circadian rhythm analysis (NPCRA) implemented in the software (Sleep Analysis v7). The following outcome parameters were assessed: intra-daily variability (IV), inter-daily stability (IS), the onset time of a period of 10 consecutive hours with greatest activity (M10) which normally occurs during daytime, as well as the onset time of the 5 consecutive hours with least activity (L5), i.e. during sleep. The absolute activity amplitude is indicated in arbitrary units whereas the relative amplitude reflects the ratio of M10/L5 amplitudes. In general, a greater IV, a lower IS as well as lower relative amplitude depict greater rest-activity fragmentation across several days. The sleep variables were derived from rest-activity cycles and analyzed by the sleep analysis feature of the same software.

### Statistics

The Software Package SAS (SAS Institute Inc., Cary, NC, USA; v 9.3) was used for statistical analyses. For all within-between subject analyses, linear mixed model analysis with the factors STATION (Halley VI vs. Concordia), and the repeated factor TIME (months 1–7; please see next paragraph for details) and LIGHT STIMULUS (blue light stimuli scotopic: 1–4; red light stimuli photopic: 1–5; bright blue scotopic and photopic: 1–2) were used on relative or standardized data (z-scores, see below). AGE (all analyses) and SEX (only for circadian and sleep analysis) were added as covariates, both employed on dichotomized data (from median splitting), if not otherwise stated in the text. For pupil analyses, the respective first BL pupil size (i.e. BL pupil size immediately following light or dark adaptation, respectively) was added as covariate to the analysis. Post-hoc tests were performed using the Tukey-Kramer test (adjusted for multiple comparisons), or the effect slices from least-square means (adjusted for multiple comparisons by the false discovery rate procedure; FDR).

In order to statistically compare the (same) time range for both stations, the data was collapsed into monthly bins (=28 days if not otherwise stated in the text) as following: the first month prior to the last sunrise (April), the three months during the time without direct sunlight (May-July) and the three months after the direct sunlight came back (August-October). This resulted in 7 monthly bins. The last day with direct sunlight was 2 days apart between both stations and matched approximately calendar months.

In addition to this, for the pupil protocol, the averages of all participants per station and all available weeks were subjected to a non-linear curve-fitting in order to assess the time of the maximum response per station. For the non-linear curve fitting a (damped) sine curve was used [formula: f = a * exp (−x/c) * sin (pi * (x-x0)/b; Sigma Plot, v11.0] and adjusted R^2^ values are reported. The non-linear curve-fitting was applied on standardized data by calculating z-scores (z = x − μ/SD; where x is the raw score of the data; μ indicates the mean and SD the standard deviation). The time at maximum response for each pupil protocol was determined on the fitted regression line. Statistical associations between the pupil parameters and the circadian and sleep data were performed by using a Spearman Correlation on average variables per participant and month.

## Electronic supplementary material

Supplementary Information
