# Supplementary material for: Impact of long-term daylight deprivation on retinal light sensitivity, circadian rhythms and sleep during the Antarctic winter
Source: Sci Rep. 2018 Nov 1;8:16185. doi: 10.1038/s41598-018-33450-7 (PMC6212492; doi:10.1038/s41598-018-33450-7)
Supplement: Supplementary file 1 — Supplementary Information [file 41598_2018_33450_MOESM1_ESM.pdf]

Supplemental Material

Impact of long-term daylight deprivation on retinal light sensitivity, circadian rhythms and sleep during the Antarctic winter

Kawasaki A[1], Wisniewski S[2,5], Healey B[3], Pattyn N[4], Kunz D[2,5,6], Basner M[7], Münch M[2,5,8]

[1]University of Lausanne, Hôpital Ophtalmique Jules Gonin, Fondation Asile des aveugles Lausanne, Switzerland; [2]Charité Universitätsmedizin Berlin, corporate member of Freie Universität Berlin, Humboldt-Universität zu Berlin, and Berlin Institute of Health, Laboratory of Chronobiology, Berlin, Germany, Institute of Physiology; [3]National Health Service UK; [4]Vrije Universiteit Brussel, Department of Human Physiology & Royal Military Academy, VIPER Research Unit, Brussels, Belgium; [5]St. Hedwig-Krankenhaus, Berlin, Germany; [6]Intellux GmbH, Berlin, Germany; [7]University of Pennsylvania; Department of Psychiatry, Perelman School of Medicine, Philadelphia, USA; [8]Charité Universitätsmedizin Berlin, corporate member of Freie Universität Berlin, Humboldt-Universität, Institute of Medical Immunology, Berlin, Germany

Content Supplemental Material:

- Pre- and post-mission training
- Electrical lighting conditions on Halley VI and Concordia
- Light stimulus protocols
- Baseline pupil size
- Figure S1: day- and twilight durations
- Supplemental Table 1 and 2

Pre- and post-mission training

During the pre-mission training in Europe, participants underwent a screening eye fexamination which included: complete ocular history, acuity testing using Jaeger reading card, color vision testing using Ishihara book, direct funduscopic examination, confrontation visual field testing and clinical evaluation of the pupil light reflex of each eye. For 20 participants, this screening examination was performed by one of the investigators (AK). One Halley VI crew member was examined by his local ophthalmic specialist. For two crew members from Concordia, the eye examination was performed by the medical doctor on the mission because both participants joined the crew after the pre-mission training. One Halley VI crew member did not undergo the screening eye examination because he joined the crew after the start of the mission. The results of the screening eye examination were normal for all participants except two members of the Halley VI crew who had reduced acuity in one eye. For one participant, this was due to longstanding amblyopia from childhood anisometropia and for the other participant, the reduced acuity was due to cataract. For these two participants, the left eye was used for pupil testing during the mission.

During the pre-mission training, baseline pupil recordings (see below) were obtained for 10 Halley VI and 11 Concordia participants. Also during the pre-mission training, participants were asked to complete four validated questionnaires in their native language of English, French or Italian: the Munich-Chronotype Questionnaire (MCTQ), the Horne-Ostberg Morning/Evening Questionnaire (HO), the Pittsburgh Sleep Quality Index (PSQI) and the Seasonal Pattern Assessment Questionnaire (SPAQ). Completed questionnaires were obtained from 11 out of 12 Halley VI participants and 12 out of 13 Concordia participants (except for the PSQI : 11 out of 13 participants). None of the participants was excluded based on questionnaire scores, the results are summarized on Table S1 of the supplemental material. There was no statistical difference for age or any of the questionnaire results between the two crews (two-sided t-test: $p > 0.36$; Table S2).

Electrical lighting conditions on Halley VI and Concordia

Artificial light levels (produced by mostly ceiling mounted fluorescent tubes) were not continuously monitored but measured once during the mission on both stations. The measurements (with electrical light only) were taken in all rooms, in four different directions and averaged for a sitting and a standing position on a vertical plane at the approximate eye level of an observer. Median illuminance at the Concordia station was 116 ± 148 lux (SD; range 10 lux - 783 lux) and at Halley VI was 425 ± 297 (SD; 3 - 1243 lux; t-test between both stations: $p < 0.05$). During the overwinter mission, all crew members had daily working routines with free days interleaved.

Participants were not restricted to staying indoors. However, given the extreme weather conditions, time outside was generally limited.

Light stimulus protocols

Light stimuli were generated by four LEDs having narrow bandwidth (470 nm blue, 633 nm red) and presented through a diffuser at a focal distance of 36 mm. The light intensities ranged from -4.0 to 2.23 log cd/m2. The angle of illumination was 46°x 54°. All light stimuli in this study were 1 s in duration. The mean pupil size in darkness during 0.25 s before each light stimulus was reported as baseline pupil size (BL). In order to account for any changes in baseline pupil size during the testing protocol, pupil responses [maximum contraction amplitude (CA) and post-illumination pupil response (PIPR)] were expressed relative to the corresponding pre-stimulus baseline pupil size. The first test sequence was performed under conditions of dark adaptation (0 lux).

Participants wore a sleep mask on both eyes and were instructed to close their eyes for 10 minutes. Thereafter the mask was removed (eyes still closed) as the rubber eyepiece of the pupilometer was placed over the tested eye and the un-tested eye was manually occluded. Once occlusion with the eyepiece was ensured, the participant was asked to open the eyes and the pupil test was initiated. The pupil was recorded in darkness for 3 s and then a sequence of 5 blue light stimuli was presented. The first light stimulus was very dim with intensity at -4.0 log cd/m^2 and the next 3 stimulus intensities increased in approximately half-log unit steps to -2.4 log cd/m^2. For purposes of analysis, these 4 dim blue lights comprised the 'scotopic protocol' which was biased to favour rod activation over the other photoreceptors and thus considered a rod-weighted stimulus protocol. The fifth light stimulus in this dark-adapted test sequence was a much brighter blue light stimulus (2.23 log cd/m^2) and was not included in the analysis considered 'scotopic protocol'. This last bright blue light stimulus was analysed as part of the 'bright blue light protocol' which was intended to evaluate the post-illumination pupil response after dark adaptation (PIPR; see below).

The second test sequence was the photopic protocol performed following light-adaption (room light for 10 min). The room light in the testing room was 566 lux (in a vertical direction at eye level for a sitting person) at Halley VI and 265 lux at Concordia. The pupil was recorded continuously for 3 s of darkness and then in response to a series of five red lights. The first four stimulus intensities increased in half log-unit steps (from 0 to 1.5 log cd/m^2) and the fifth stimulus intensity was 2.6 log cd/m^2. An inter-stimulus interval of 3 s permitted the pupil to return to baseline size before the next stimulus. All 5 red light stimuli comprised the 'photopic protocol' which was predominantly weighted to M and L cone activation.

The third test sequence was performed after the photopic protocol under the light conditions described above. This sequence used 2 blue bright light stimuli (1.75 log cd/m^2 and 2.23

log cd/m^2) with a prolonged inter-stimulus interval of 15 s in order to record the post-illumination pupillary dynamics. For the post-recording analysis, these two stimuli were combined with the bright blue light stimulus recorded under dark adapted conditions to comprise the 'bright blue light protocol'. This protocol was aimed to evaluate mainly melanopsin contribution to the pupil response which is best differentiated from the influence of rods and cones by examining the post-illumination pupil response (PIPR; [1]).

Baseline pupil sizes

The baseline (BL) pupil sizes were analyzed for each protocol with the factor LIGHT STIMULUS (4 different light stimuli for the scotopic protocol, 5 different light stimuli for the photopic protocol, 1 light stimulus for the dark adapted, and 2 different light stimuli for the light adapted bright blue light protocol), the repeated factor TIME (April - October) and STATION (Halley VI vs. Concordia). The factor AGE was added in a separate analysis to the model (see below).

For BL pupil sizes of the scotopic protocol, there was a main effect of TIME such that dark adapted BL pupil sizes were significantly smaller in the first two months (April, still with direct sunlight, and May) when compared to all other months ($F_{6, 554} = 17.86$; $p < 0.0001$) and it was largest in the last month (October), compared to all other months. The difference between the first and the last month was on average 0.49 cm. BL pupil sizes became, on average, 0.23 cm smaller between the first and the last light stimulus (main effect of LIGHT STIMULUS; $_{3, 554} = 25.59$; $p < 0.0001$; Table S1). There was no statistical significant difference in BL pupil size between the two stations at any month (post-hoc tests from significant interaction STATION x TIME; $p > 0.6$), and no other interactions with the factors STATION, TIME or LIGHT STIMULUS ($p > 0.15$).

The BL pupil sizes for the photopic red light stimuli were significantly smaller in the first two months (April-May) compared to all other months (main effect of TIME; $F_{6, 703} = 97.17$; $p < 0.0001$). There was no difference between both stations at any month for post-hoc tests from significant interaction STATION x TIME, $p > 0.18$) and no other statistical significant interactions with STATION, TIME or LIGHT STIMULUS. With increasing light intensity, the BL pupil size got smaller by 0.21 cm between the first and the last light stimulus (main effect of LIGHT STIMULUS; $F_{4, 703} = 45.82$; $p < 0.0001$).

The dark adapted baseline pupil (BL) before the bright blue stimulus was significantly larger in the last month (October) than April-June and August (main effect of TIME; $F_{6, 122} = 5.43$; $p < 0.0001$), without a significant difference between stations and no significant interactions with TIME or LIGHT STIMULUS. The light adapted baseline pupil sizes for the bright blue light protocol were significantly smaller in the first month (April; i.e. the last month with direct sunlight) when compared to all other months (main effect of TIME; $F_{2, 267} = 39.83$; $p < 0.0001$), and was also smaller in May (first month

without direct sunlight) compared to the last month (October). The crew on Halley VI had also significantly smaller pupil BL sizes in April compared to those of the Concordia crew (TIME x STATION; $F_{2, 267}$ = 27.55; p <0.0001; Halley VI: 4.71 ± 0.79 mm and Concordia: 5.56 ± 1.0 mm). There was also a significant difference between the BL pupil size before the first and the second bright blue light stimulus of 0.3 mm (main effect of LIGHT STIMULUS; $F_{1, 267}$ = 74.7; p <0.0001), without any interaction with TIME or STATION. When the factor AGE was added as covariate to the above analyses, the older subgroup had in all protocols smaller pupil sizes than the younger subgroup (AGE: p <0.05).

Figure S1:

Annual distribution of direct sunlight, civil twilight and nautical/astronomical twilight averaged for both stations and the data collection period (2015).

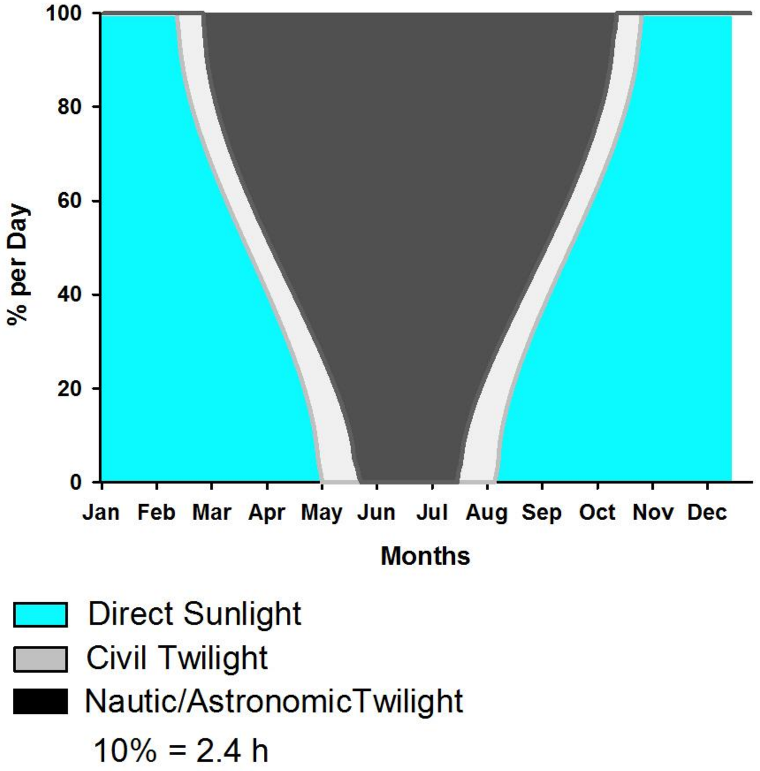

The graph shows the % of direct sunlight (cyan), civil twilight (grey; i.e. the geometric center of the sun is at most 6° below the horizon), and nautical/astronomical twilight (black; nautical twilight: the sun is between 6° and 12° below the horizon; astronomical twilight: the sun is between 12°-18° below the horizon) for the year 2015 at the Antarctica. 10 % equals to 2.4 h. The information for sunshine duration and twilight derived for Concordia from (both assessed on Jan. 2. 2018): https://www.timeanddate.com/sun/antarctica/concordia-station?month=1&year=2015; and for Halley VI from: http://dateandtime.info/de/citysunrisesunset.php?id=6620756&month=12&year=2015

Environmental illuminance: Illuminance with sunlight and clear sky: 20´000-100´000 lux (Source: https://en.wikipedia.org/wiki/Daylight#Intensity_in_different_conditions); twilight illuminance: …' *During a clear evening's civil twilight, horizontal illuminance decreases from:~585–410 lux to ~3.5–2 lux; during a clear evening's nautical twilight, horizontal illuminance decreases from: ~3.5–2 lux to ~0.008 lux; astronomical twilight, horizontal illuminance due to scattered sunlight decreases from ~0.008 lux to ~6 × 10^{-4} lux.'…* (Source: http://glossary.ametsoc.org/wiki/Civil_twilight).

Table S1: Baseline pupil sizes (in mm)

Month	Scotopic (mm)	SD	Photopic (mm)	SD	Dark Adapted Bright Blue Stimuli (mm)	SD	Light Adapted Bright Blue Stimuli (mm)	SD
1 (April)	6.17	1.01	5.00	0.87	5.91	0.94	5.17	0.99
2 (May)	6.24	1.00	5.38	0.84	5.96	0.96	5.64	0.89
3 (June)	6.36	0.91	5.50	0.90	6.05	0.88	5.74	0.91
4 (July)	6.39	0.94	5.57	0.85	6.12	0.90	5.79	0.86
5 (Aug)	6.41	0.88	5.54	0.86	6.09	0.91	5.76	0.83
6 (Sep)	6.42	0.93	5.60	0.81	6.15	0.96	5.86	0.87
7 (Oct)	6.67	0.80	5.75	0.78	6.42	0.82	6.05	0.84

Table S1 shows the averaged baseline (BL) pupil sizes in mm (mean, SD) per month for each protocol: scotopic (mean of 4 BL pupil sizes before weak blue light stimuli); photopic (mean of 5 BL pupil sizes before bright red light stimuli); dark and light adapted BL pupil sizes before bright blue light stimuli (the latter: mean of BL pupil sizes to both bright blue light stimuli). For statistical differences per protocol between months see text.

Table S2: Questionnaire results for both stations

	Age (yrs)	Sex	PSQI	MCTQ	HO	SPAQ
Halley VI	33.7 (12)	4f/8m	4.55	4.38	51.18	7.45
SD	11.2		2.25	0.69	9.12	4.44
Concordia	34.6 (13)	3f/10m	4.73	4.36	54.38	7.17
SD	11.0		2.33	0.96	7.36	4.00
Both	34.1	7f/18m	4.64	4.37	52.78	7.31
SD	0.6		2.29	0.83	8.24	4.20

Table S2 shows the questionnaire results which were completed before the mission for most participants (see also text in the Supplemental Material). The mean values ± SD are shown per station for age (n=25) as well as the Pittsburgh Sleep Quality Index (PSQI); the Munich Chronotype Questionnaire [MCTQ (MSF-sc); corrected for sleep duration on free days]; the Horne-Ostberg Morningness-Eveningness Questionnaire (HO) and the Seasonal Pattern Assessment Questionnaire (SPAQ). For Halley VI a total of 11 (out of 12) and for Concordia 12 (out of 13; and for the PSQI: 11) participants completed the PSQI, MCTQ, HO and SPAQ Questionnaires.

Reference

1 Park, J. *et al.* Toward a clinical protocol for assessing rod, cone, and melanopsin contributions to the human pupil response. *Invest Ophthalmol Vis Sci* 52, 6624-6635 (2011).
